# Optimizing health services for young children in poverty: enhanced collaboration between Early Head Start and pediatric health care

**DOI:** 10.3389/fpubh.2024.1297889

**Published:** 2024-02-14

**Authors:** Diane M. Horm, Holly E. Brophy-Herb, Carla A. Peterson

**Affiliations:** ^1^Early Childhood Education Institute, University of Oklahoma-Tulsa, Tulsa, OK, United States; ^2^Department of Human Development and Family Studies, College of Social Science, Michigan State University, East Lansing, MI, United States; ^3^Department of Human Development and Family Studies, College of Human Sciences, Iowa State University, Ames, IA, United States

**Keywords:** infant, child health services accessibility, interinstitutional relations, poverty, Early Head Start, cross-system collaboration

## Abstract

Given the importance of health to educational outcomes, and education to concurrent and future health, cross-systems approaches, such as the Whole School, Whole Community, Whole Child (WSCC) framework, seek to enhance services typically in K-12 settings. A major gap exists in cross-systems links with early care and education serving children birth to age 5. Both pediatric health systems and early family and child support programs, such as Early Head Start (EHS) and Head Start (HS), seek to promote and optimize the health and wellbeing of infants, toddlers, preschoolers, and their families. Despite shared goals, both EHS/HS and pediatric health providers often experience challenges in reaching and serving the children most in need, and in addressing existing disparities and inequities in services. This paper focuses on infant/toddler services because high-quality services in the earliest years yield large and lasting developmental impacts. Stronger partnerships among pedicatric health systems and EHS programs serving infants and toddlers could better facilitate the health and wellbeing of young children and enhance family strengths and resilience through increased, more intentional collaboration. Specific strategies recommended include strengthening training and professional development across service platforms to increase shared knowledge and terminology, increasing access to screening and services, strengthening infrastructure and shared information, enhancing integration of services, acknowledging and disrupting racism, and accessing available funding and resources. Recommendations, including research-based examples, are offered to prompt innovations best fitting community needs and resources.

## Introduction

Past research documents bidirectional associations between health and educational outcomes and acknowledges that health conditions, disabilities, and unhealthy behaviors affect educational outcomes (1, 2). A growing body of research illustrates the influence of education on health (3, 4) and suggests the effects of educational programs on health start early and have lasting impacts. Fortunately, these effects are malleable; programs have been shown to produce significant, positive impacts. For example, randomized long-term studies of high-quality early education programs designed for infants, toddlers, and preschoolers show improved health and fewer risky health behaviors at ages 21 (5), 30 and 40 (6, 7). Thus, Chiang et al. (3) observation that despite these overt links, “the health and education sectors have, for the most part, grown, developed, and established their influence independent of each other” (p. 775) is troubling. Even more perplexing, health and education professionals focus on similar issues, often with the same children, in the same neighborhoods, yet work in parallel often with minimal collaboration (3, 8).

Given the important connections between health and educational outcomes, health promotion initiatives have been developed, typically drawing on multiple disciplines and recognizing the role of different systems (e.g., home, school, neighborhood). Building on earlier models, the Whole School, Whole Community, Whole Child (WSCC) framework is a health promotion approach advanced by the Centers for Disease Control and Prevention (8) for integrating health and education in schools (3, 9). The WSCC model comprehensively assesses strengths and needs and highlights common goals of both health and education to prompt adoption of a whole child approach (8).

The WSCC model was designed primarily for K-12 settings. While WSCC descriptions include many community organizations, links to early care and education (ECE) serving children birth to age 5 appear to be missing. Research summarizing the long-term benefits of ECE on later outcomes demonstrates school health promotion programs and ECE share common goals. Additionally, a whole child approach and comprehensive service delivery model are distinguishing features of many ECE programs, including Early Head Start (EHS) and Head Start (HS), federally-funded programs serving children birth to age 5 and their families living in the context of poverty across all U.S. states and territories.

Although EHS/HS strive to deliver comprehensive services and have experienced successes integrating education with health and other services (10), stronger partnerships among pediatric health systems, school health promotion, and ECE could further strengthen services and optimize outcomes. This paper calls for greater collaboration across these systems.

We focus on the youngest children because a growing body of research suggests that ECE programs for infants offer the largest and longest-lasting impacts (6, 11). Likewise, we highlight young children and their families living in poverty because they face multiple stressors known to impede both educational and health outcomes. In response, pediatric care, public health, and comprehensive programs designed for young children seek to address children's myriad needs adopting a prevention framework (8). These systems note challenges in reaching those most in need and successfully addressing existing inequities in service access and receipt. For example, vaccination rates often lag for infants and toddlers among impoverished families without access to insurance (12). Similarly, EHS served just 11% of eligible children in 2021 (13).

We first describe EHS because, although launched in 1995, it is less familiar than its companion program, HS, that has served preschool-aged children since 1965. We then offer strategies to strengthen collaboration across EHS and pediatric health systems.

## Early Head Start

EHS provides an array of services to pregnant women and families with children under age 3 living in poverty (14), extending the comprehensive services offered through HS to infants and toddlers. In 2020, EHS and HS together served more than 839,000 children across the US; 30% were under age 3 (15). EHS programs follow the Head Start Program Performance Standards (HSPPS) stipulating provision of high-quality, comprehensive child development services through home visits, childcare, case management, parenting education, health care and referrals, and family support (16, 17). They must also meet Early and Periodic Screening, Diagnosis, and Treatment (EPSDT) requirements (16). The goal of comprehensive services, an EHS “cornerstone” (18), is to promote the health and wellbeing of children and support families as caregivers (19). Thus, comprehensive EHS services are designed to address the needs of infants, toddlers, and families including facilitating health, mental health, and early intervention (EI) services. See the following website for additional information about EHS: https://eclkc.ohs.acf.hhs.gov/programs/article/about-early-head-start-program.

## Theoretical and practical foundations

In ECE, existing models similar to WSCC are used. For example, delivery of comprehensive services across systems is well-aligned with contemporary models of child and family development, including early relational health (ERH). ERH highlights two important considerations. First, wellbeing is defined comprehensively to include children's physical health (e.g., receipt of health care, well-child visits), developmental health (e.g., optimal development across domains), and relational health (e.g., the child's positive relationships with parents/caregivers, teachers) (20). Second, ERH frameworks (21) highlight essential relationships across three, interrelated systems: (1) primary relationships between caregivers and children (e.g., parent-child/teacher-child relationships); (2) secondary relationships among caregivers and service providers (e.g., healthcare providers, social workers, ECE providers); and (3) tertiary relationships among larger systems impacting children's wellbeing, including communication and infrastructure to support coordination among systems (e.g., ECE programs including EHS, primary healthcare, and EI).

ERH, the common framework driving systems thinking across birth to age 5 services, shares similarities with WSCC's systemic approach. ERH, similar to WSCC with older children, provides a framework supporting integration of children's physical, developmental, and mental health needs and services. Practically speaking, ERH models offer the most robust framework for early support/intervention given the strong influence the health of infants and toddlers has across developmental domains (22). Calls to provide integrated services advocated by ERH and WSCC are crucial for the youngest children; while public school systems serve over 91% of children K-Grade 12 (23), U.S. ECE is fragmented with many children and families underserved.

## Health, mental health, and early intervention services in EHS

The HSPPS (16) detail requirements for EHS/HS comprehensive services (Table 1 provides details). Expanding collaboration between EHS and health care is well-aligned with EHS and pediatric health goals. Further, growing recognition that racial and economic inequities start well before birth (13) is a clarion call that inequities must be acknowledged and attention focused on creating practices, policies, and systems that achieve equity in service access and receipt. Next, we summarize how EHS addresses health, mental health, and EI service needs as background for discussion on how EHS/HS and pediatric services might expand collaborations.

**Table 1:** Service needs/goals and associated EHS mandates related to health, mental health, and EI.

**Service Needs/Goals**	**EHS mandates as stipulated in HSPPS (16, 17)**
Increasing full-term births	§1302.46 Addresses family support services for health, nutrition, and mental health The following opportunities must be offered: opportunities to learn about healthy pregnancy and postpartum care, as appropriate, including breastfeeding support and treatment options for parental mental health or substance abuse problems, including perinatal depression.
Increasing breastfeeding	§1302.44 Child nutrition Requirements include promoting breastfeeding, including providing facilities to properly store and handle breast milk and making accommodations, as necessary, for mothers who wish to breastfeed during program hours, and if necessary, provide referrals to lactation consultants or counselors.
Increasing safe environments	§1302.47 Safety practices Programs must develop and implement a system of management, including ongoing training, oversight, correction and continuous improvement that includes policies and practices to ensure all facilities, equipment and materials, background checks, safety training, safety and hygiene practices and administrative safety procedures are adequate to ensure child safety.
Increasing rates of immunizations, health insurance coverage, and connection to a medical home	§1302.42 Child health status and care EHS must ensure up to date health status and records and ongoing care.
Ensuring families have needed health-related supplies	§1302.42 Child health status and care Programs are to use funds to provide diapers and formula during the EHS program day.
Supporting child nutrition	§1302.44 Child nutrition Relative to nutrition services, EHS programs must: • Feed infants and toddlers according to their individual developmental readiness and feeding skills as recommended in USDA requirements • Ensure infants and young toddlers are fed on demand to the extent possible; and • Ensure bottle-fed infants are never laid down to sleep with a bottle.
Supporting children with behavioral concerns	§1302.17 Suspension and expulsion Children cannot be expelled or disenrolled because of behavior. Steps must be taken to address behavioral concerns and support the child in the program, including working with mental health consultants, identifying services and supports addressed in section 504 of the Rehabilitation Act, including attention to definitions of disability in 29 U.S.C. 705(9)(b) of the Rehabilitation Act, carrying out an established individualized family service plan (IFSP) or individualized education program (IEP), or taking steps to determine eligibility for services.
Providing child mental health services	§1302.45 Child mental health and social and emotional wellbeing EHS must include:(a) Wellness promotion (b) Mental health consultants
	§1302.46 Family support services for health, nutrition, and mental health EHS must discuss with staff and identify issues related to child mental health and social and emotional well-being, including observation and any concerns about their child's mental health, typical and atypical behavior and development, and how to appropriately response to their child and promote their child's social and emotional development.
Promoting healthy parent-child relationships	§1302.34 Parent and family engagement in education and child development activities §1302.35 Education in home based programs EHS must develop and deliver strategies and activities that promote parents' ability to support the child's cognitive, social, emotional, language, literacy, and physical development.
Promoting parental mental health	§1302.46 Family support services for health, nutrition, and mental health EHS must provide opportunities for families to learn about healthy pregnancy and postpartum, including breastfeeding support and treatment options for parental mental health, including perinatal depression, or substance use.
	§1302.53 Community partnership and coordination with other early childhood education programs Subpart H- Services to Enrolled Pregnant Women including 1302.81 prenatal and postpartum information, education, and services EHS must build collaborative partnerships with other community organizations, which may include, for example, health care providers attending to child and adult mental health (e.g., addressing postpartum depressive symptoms) and domestic violence prevention and support service organizations.
Serving children with disabilities	§1302.60 to 1302.63 Additional services for children EHS must promote full participation in program services, additional services for children and parents and coordination and collaboration with agencies responsible for implementing the Individuals with Disabilities Act (IDEA). Also, programs must provide additional services for children with an IFSP or IEP. Programs must work closely with other service providers and plan and implement transition services for children leaving EHS, and plan appropriate transition services for children with an IFSP.
Utilizing individualized instructional practices	§1302.60 Full participation and least restrictive environment Full participation in program activities and services must be delivered in the least restrictive environment.
Providing screening and assessment	§1302.33 Child screenings and assessment Child screenings and assessments, including information on screening and assessment procedures, characteristics of screenings and assessment tools, and restrictions on the use and purposes of screening and assessment data (e.g., data cannot be used for exclude children from the program and cannot be used to sanction or reward children, teachers, or other staff) must be provided.

### Health services

Rates of immunizations and insurance coverage are important health indicators that vary across states and sociodemographic groups. EHS programs work successfully with families to increase access with rates of immunization (94% to 96%) and health insurance (95% to 97%) increasing over the 2018–19 HS program year (24). EHS also shows favorable impacts on increased breastfeeding and other health promoting practices (22, 25). These indicators reflect, in part, the resources and care a medical home provides. Unfortunately, most states report only about 50% of parents have a medical home for their infants and toddlers, with rates varying widely by race and ethnicity (13). Participation in EHS/HS boosts rates of connection to medical (95% to 97%) and dental homes (82% to 90%) (24) underscoring the potential to further increase service access and child wellbeing via enhanced EHS-pediatric health collaborations.

### Mental health services

One out of every six children, ages 2 to 8, has a diagnosed mental health, behavioral, or developmental disorder (26). Mental health concerns are disproportionately high among children from impoverished contexts, where nearly 20% of children experience a mental, behavioral, and/or developmental disorder (27), and among children from ethnic-racial minorities (28). Unfortunately, access to full mental health services lags behind needs (29). For example, about 78% of children with depression receive treatment but only 54% of children with behavioral disorders receive treatment (30). Among children experiencing poverty, fewer than 15% receive needed mental health services (27), and Black children are less likely than White children to receive treatment (31). Fortunately, EHS/HS are effective vehicles for linking children and families with mental health services. HSPPS require provision of infant and early childhood mental health consultation, which is linked to reductions in children's behavioral challenges (32). Infant and early childhood mental health consultants partner with EHS/HS teachers to promote children's mental health via classroom practices and with case managers, teachers, and families to address children's behavioral concerns (33). Starting early is effective with children enrolled in HS at age 3 more likely to receive mental health treatment than those enrolled at age 4 (31).

### Early intervention services

EI exists at the nexus of health, education, and social services by both the nature of services and legislative mandate. EI services are available to every U.S. child, birth to age 3, who has a developmental delay or diagnosed condition with a high probability of resulting delay. Services can include special instruction, psychological services, speech and language therapy, physical or occupational therapy, nursing and nutrition services, hearing and vision services, social work services, transportation, and assistive technology. More children are identified as eligible for EI as they age. Approximately 3.5% of children under age 2 received EI services and ~6.8% of children ages 3 to 5 received early childhood special education services in 2018 (34). However, ethnic-racial disparities are evident. For example, infants (age 9 months) receive EI services at approximately the same rate, but Black toddlers (age 24 months) are five times less likely to receive EI services than their White counterparts (35). Delays in identification are problematic because EI services can influence long-term success for children and families (36). EHS provides frequent developmental screening and supports families navigating the EI system; those enrolled in EHS (5.7%) were more likely to receive Part C services than those in a control group (3.7%) (37).

## Recommendations to improve/strengthen delivery of these comprehensive services

The need to strengthen the availability and integration of services was illustrated by the COVID-19 pandemic that highlighted disparities in access and focused attention on the fragility and critical importance of U.S. early childhood “systems” (38). These lessons underscore the need to build more robust approaches that forge stronger connections across primary health care, ECE, social services, and other systems (38). Recommendations (summarized in Table 2) offered here to enhance cross-system collaboration apply to health, mental health, and EI service needs.

**Table 2:** Summary of recommendations to improve/strengthen delivery of comprehensive services to children and families.

**Recommendation**	**Examples of strategies**
Strengthen training and professional development	•Expand preservice and inservice training across professional domains (e.g., medicine, social work, early childhood education) on relational health and allied systems to expand knowledge and promote common language and understanding • Provide medical students with rotation experiences in EHS/HS and EI programs, and provide preservice early childhood specialists with field experiences in pediatric clinics
Expand service delivery models and integration of services	•Expand screenings and related service referrals • Embed pediatric care, including additional screenings in EHS/HS program sites and/or • Embed EHS/HS and EI information and referrals in pediatric settings • Study and learn from existing collaborative models
Strengthen infrastructure and shared information	•Link child health, education/care, and welfare data systems • Establish efficient and low maintenance referral systems • Expand use of navigation models to assist in service coordination, reduce barriers, and increase family access to services
Acknowledge and take steps to reduce racism and promote equity	•Increase training to build knowledge about racism • Increase awareness of implicit and explicit bias • Explore strategies (e.g., reflective supervision, consultation) as tools to create environments in which practitioners can identify and lessen biases • Examine health and education statistics by race, including working to identify barriers to access to services • Work with community constituents to set goals and measure success toward increasing racial equity in services • Ensure EHS/HS, EI, and pediatric clinics provide materials and oral communication in primary languages spoken by families
Identify, secure, and maximize resources	•Explore combined mechanisms of funding at city, regional, state, and federal levels

### Strengthen training and professional development

Cross-system collaboration calls for a paradigm shift in how systems engage with children and families and how they interact with each other (39). Strengthened professional development is needed to enhance integration. Specific recommendations include expanding preservice and inservice training on ERH and allied systems, including pediatric health and EHS, across professional domains. Discipline specific training could include preparing preservice (e.g., students in medicine, public health, social work, ECE) and front-line practitioners (e.g., home visitors, teachers, nurses, physicians) to attend to the complex interactions that impact mental health (e.g., historical trauma, systemic racism, environmental injustices) (40). Students would benefit from clinical experiences in both pediatric and EHS contexts. Incorporating information about EHS/HS and similar ECE programs into schools of medicine and public health would promote understanding and reinforce shared goals for promoting child health and wellbeing.

### Expand screening and service delivery models

Integrating screening and comprehensive services would increase access to services, strengthen relationships among systems, and streamline service provision. Enhanced screenings and greater service access are key to integration of services, which includes efforts to embed child and family support into primary pediatric care (27). A related strategy could embed mobile pediatric care into EHS/HS programs, making services more accessible to families and facilitating provision of cross system services.

EHS/HS and community partners working together to provide free screenings, could reverse the unfortunate trend of decreasing access to screenings (e.g., vision, hearing, behavioral, developmental) without unnecessary burden on any partners (29). Additional barriers to screening include lack of families' access to consistent transportation and translation services (e.g., just 24% of HS agency respondents indicated health care providers had bilingual staff) (29).

Examining successful models can help generate ideas. For example, the National Center on Early Childhood Health Wellness (41) offers a step-by-step guide to building collaborations between EHS/HS and medical homes. Importantly, collaborations may begin small between a single pediatric clinic and one EHS program with both parties sharing expertise, materials, and information; building knowledge of each other; and supporting bidirectional referrals (41).

Another example, Warm Connections, integrates IMH services in local WIC programs (42). Trained clinicians provide brief mental health and parenting supports and referrals to community resources, including more intensive community mental health referrals. In addition to service access, pilot findings suggest the program may relieve parenting distress and increase parental efficacy.

Healthy Steps by Zero to Three (43) programs partner with pediatric clinics to offer families child development information and supports. services include screenings, referrals, and facilitating families' access to other services, including care coordination and systems navigation.

### Strengthen infrastructure

Strengthening and coordinating administrative and data infrastructure is important for building working relationships and monitoring service effectiveness. Linking child health, education/care, and welfare data systems could promote improved communication and better understanding of service distribution; additionally, this could facilitate identification of service gaps. For example, linking kindergarten entrance assessments and medical records could facilitate appropriate screenings/referrals and target service areas (44). Cross sector data integration would enable annual reviews of service delivery, coordination, and gaps. A randomized trial linking child health directly with ECE services, in one urban pediatric clinic, showed a computer-generated enrollment packet sent directly to HS (referral letter, physical examination, and immunization records) from the pediatric office increased HS enrollment compared to providing parents a list of HS contacts (45). This approach resulted in increased HS attendance and increased HS direct contacts with families (46).

Navigators or guides can also shore-up infrastructure. Using navigation to assist in service coordination, reduce barriers, and increase family access has begun to move from medical to social service settings. Recently, Waid et al. (47) illustrated navigation services were successfully facilitated by clinicians, peers, paraprofessionals, service teams, or via self-direction in a variety of formats (e.g., in person, telephone, online). Diaz-Linhart et al. (48) evaluated a navigator program for families in HS, who were randomized to the navigation or a usual care group. Navigators worked successfully with families to overcome barriers with families receiving navigation reporting more connections with external mental health services than parents in the usual care group.

### Acknowledge and reduce racism

The Head Start Head Start Early Childhood Learning and Knowledge Center (49) offers training designed to engage programs and professionals in conversations about racism, including recognizing and opposing racism in all forms (50), and developing anti-bias practices and policies. Trainings address levels (e.g., internalized, personally mediated, and institutionalized racism) (51) and forms of racism and implicit bias. Strategies such as reflective supervision may be effective in supporting practitioners' awareness of personal and systemic biases (52). The Annie E. Casey Foundation (53) offers a toolbox for setting goals/measuring success toward increasing racial equity, assessing the impact of policies on minoritized groups, and engaging community partners in promoting social change. Additional strategies include reporting health and education statistics by race and SES, already required for education records, to assist in identifying areas for increasing equity in access and service provision and examining current practices.

### Identify, secure, and maximize resources

Peterson et al. (44) note the importance of funding to incentivize and drive innovations. The Healthy Steps by Zero to Three (43) program is financed by combining multiple funding streams including redistribution of health system and graduate medical education funds, reallocation of city/county/state/local/federal supports (e.g., tobacco and cannabis sales), and philanthropic organizations. given that collaborations may begin small, initial efforts may require minimal financial resources, providing time to collect pilot data to fuel more expansive funding applications. Another strategy is to identify and target funding to prompt innovative, transdisciplinary work outside the typical health and education siloes including potential federal funding supporting coordination efforts. For example, federal funds appropriated to the U.S. department of health and human services and jointly administered with the U.S. department of education provided preschool development grants birth-to-five, for which states could apply, to increase access to high-quality ECE. importantly, these funds could also be used to address coordination and integration of services across systems.

Other resources include conducting SWOT analyses (Strengths, Weaknesses, Opportunities, and Threats) (54), which can assist collaborating agencies in highlighting and leveraging strengths, addressing weakness, identifying opportunities, and minimizing threats to their collaborative efforts. The SWOT approach facilitates strategic planning across collaborating agencies/systems to optimize health services for young children in poverty (55).

## Conclusions

Public health, pediatric healthcare, and EHS share a mission to enhance the wellbeing of young children, particularly those at developmental risk. Yet, each fails to reach all children and misses many of the most vulnerable. Employing comprehensive relational health models, emphasizing the interrelatedness of children's physical, mental, and developmental health, prompts new opportunities for collaborative partnerships between pediatric health systems and EHS to strengthen provision of comprehensive services. Finally, examples of strategies and tools were shared to inspire the development, implementation, and maintenance of collaborative comprehensive service models.

## Data availability statement

The original contributions presented in the study are included in the article/supplementary material, further inquiries can be directed to the corresponding author.

## Author contributions

DH: Conceptualization, Writing – original draft, Writing – review & editing. HB-H: Conceptualization, Writing – original draft, Writing – review & editing. CP: Conceptualization, Writing – original draft, Writing – review & editing.
